# Booster vaccination is required to elicit and maintain COVID-19 vaccine-induced immunity in SIV-infected macaques

**DOI:** 10.1080/22221751.2022.2136538

**Published:** 2023-03-01

**Authors:** Pingchao Li, Qian Wang, Yizi He, Chenchen Yang, Zhengyuan Zhang, Zijian Liu, Bo Liu, Li Yin, Yilan Cui, Peiyu Hu, Yichu Liu, Pingqian Zheng, Wei Wang, Linbing Qu, Caijun Sun, Suhua Guan, Liqiang Feng, Ling Chen

**Affiliations:** a State Key Laboratory of Respiratory Disease, Guangdong Laboratory of Computational Biomedicine, Guangzhou Institutes of Biomedicine and Health (GIBH), Chinese Academy of Sciences, Guangzhou, People’s Republic of China; b State Key Laboratory of Respiratory Disease, Guangzhou Institute of Respiratory Health, the First Affiliated Hospital of Guangzhou Medical University, Guangzhou, People’s Republic of China; c Guangzhou Laboratory & Bioland Laboratory, Guangzhou, People’s Republic of China; d Guangzhou nBiomed Ltd., Guangzhou, People’s Republic of China; e University of Chinese Academy of Sciences, Beijing, People’s Republic of China

**Keywords:** COVID-19 vaccine, immunogenicity, immunodeficiency, HIV, SIV infection, rhesus macaques

## Abstract

Prolonged infection and possible evolution of SARS-CoV-2 in patients living with uncontrolled HIV-1 infection highlight the importance of an effective vaccination regimen, yet the immunogenicity of COVID-19 vaccines and predictive immune biomarkers have not been well investigated. Herein, we report that the magnitude and persistence of antibody and cell-mediated immunity (CMI) elicited by an Ad5-vectored COVID-19 vaccine are impaired in SIV-infected macaques with high viral loads (> 10^5^ genome copies per ml plasma, SIV^hi^) but not in macaques with low viral loads (< 10^5^, SIV^low^). After a second vaccination, the immune responses are robustly enhanced in all uninfected and SIV^low^ macaques. These responses also show a moderate increase in 70% SIV^hi^ macaques but decline sharply soon after. Further analysis reveals that decreased antibody and CMI responses are associated with reduced circulating follicular helper T cell (TFH) counts and aberrant CD4/CD8 ratios, respectively, indicating that dysregulation of CD4^+^ T cells by SIV infection impairs the COVID-19 vaccine-induced immunity. Ad5-vectored COVID-19 vaccine shows no impact on SIV loads or SIV-specific CMI responses. Our study underscores the necessity of frequent booster vaccinations in HIV-infected patients and provides indicative biomarkers for predicting vaccination effectiveness in these patients.

## Introduction

The rapid spread of severe acute respiratory syndrome coronavirus 2 (SARS-CoV-2), the causative agent of coronavirus disease 2019 (COVID-19), has posed a huge threat to global public health [1]. While several COVID-19 vaccines have been available, their immunogenicity remains poorly understood in immunocompromised people living with human immunodeficiency virus (PLWH) [2,3]. Several vaccines have been reported to be safe and immunogenic in PLWH well controlled on antiretroviral treatment (ART) [4–6]. However, in a significant proportion of PLWH, viral replication cannot be effectively suppressed due to difficulties in accessing ART, poor adherence, or ART failure [7]. Uncontrolled HIV-1 replication leads to a gradual loss of CD4^+^ T cells, which ultimately causes immunodeficiency and immunosenescence and drives multi-morbidities [8]. Anti-viral immune responses may be impaired in these PLWH, leading to prolonged infection and delayed clearance of SARS-CoV-2 and thus facilitating viral shedding and evolution of new variants [9–11]. Of note, there is speculation that the emergence of the Omicron variant of concern (VOC) may occur in PLWH [11]. Thus, building effective immune barriers against SARS-CoV-2 in PLWH is essential for protecting PLWH and controlling the COVID-19 pandemic.

The entry of SARS-CoV-2 into host cells is initiated by the interaction between the receptor-binding domain (RBD) of viral Spike (S) protein and host receptor angiotensin-converting enzyme 2 (ACE2) [12]. A major goal of current vaccines is to elicit neutralizing antibodies (NAbs) that can block or destroy this interaction [13]. CD4^+^ T cell populations, especially the follicular helper T cell (TFH) subset, are critical for generating high-affinity antibodies and long-lived memory B cells [14]. TFH cells have been recognized as the dominant targets and major reservoirs of HIV-1 [15,16]. Their functions may be progressively damaged in the context of continuous HIV-1 replication [17]. Without proper help from TFH cells, the generation and durability of antibody responses against SARS-CoV-2, either by natural infection or vaccination, may be impaired [18]. In addition, CD4^+^ T cells contribute to viral clearance via promoting the generation of CD8^+^ T cells, mediating direct cytolytic activity, and secreting anti-viral cytokines [19]. Along with the emergence of a series of VOCs, such as Beta, Delta, and Omicron [20], whether a COVID-19 vaccine can effectively provoke immunity against SARS-CoV-2 variants in PLWH needs to be assessed.

To address these unsolved issues, we tested the immunogenicity of an adenovirus (Ad)-vectored COVID-19 vaccine, Ad5-S, that deliveries the S antigen of SARS-CoV-2 Wuhan strain [21], in rhesus macaques chronically infected with simian immunodeficiency virus (SIV). We assessed the antibody and cell-mediated immune (CMI) responses elicited by Ad5-S against the Wuhan strain and several VOCs. Key indicators that may predict the magnitude and persistence of vaccine-induced immune responses, including the baseline SIV viral loads, the counts of CD4^+^ T, TFH, central memory CD4^+^ T (Tcm), effector memory CD4^+^ T (Tem) and CD8^+^ T cells, CD4/CD8 ratios, and specific memory B cells, were evaluated in SIV-infected macaques after one and two vaccinations.

## Materials and methods

### Animals and vaccination

Chinese rhesus macaques (*Macaca mulatta*) were housed in the Landau Animal Experimental Centre, Guangdong Landau Biotechnology Co. Ltd. A total of 20 rhesus macaques were included in this study. Among them, fourteen macaques have been infected by SIVmac239 for more than 2 years and received treatment using Tenofovir (PMPA, 30 mg/kg) and Emtricitabine (FTC, 20 mg/kg). Five months before vaccination, the ART was discontinued. The SIV-infected macaques were allocated into two groups based on their baseline plasma SIV viral loads: SIV^low^ group (n = 6; mean: 3.4 log RNA copies per ml plasma; range: 2.0-4.7) and SIV^hi^ group (n = 8; mean: 6.2; range: 5.0-7.7). The rest of the 6 healthy macaques were assigned as controls (SIV^neg^). Ad5-S has been reported elsewhere [21]. All macaques were vaccinated with Ad5-S at 1 × 10^11^ vp per animal via an intramuscular injection. Fourteen weeks later, the macaques received a booster vaccination with the same dosage. At 0, 2, 4, 6, 14, 16, 18, 20, and 31 weeks after the first vaccination, serums samples and PBMCs were collected and subjected to virological and immunological analysis. Body weight, body temperature, blood biochemical indexes, and complete blood counts were monitored at the indicated time points throughout the study.

### Peptide pools

Peptide pools corresponding to the S1 and S2 subunits of the S protein of SARS-CoV-2 Wuhan strain were chemically synthesized as 20-mers with 10 amino acids overlaps between sequential peptides (Genscript, China). Peptide pools corresponding to the full-length amino acid sequences of SIVmac239 Gag and Pol were obtained through NIH HIV Reagent Programme, Division of AIDS, NIAID, NIH, contributed by DAIDS/NIAID. The SIV peptides were produced as 15-mers with 11 amino acid overlaps. All peptide pools were dissolved with dimethyl sulfoxide (DMSO, Sigma) to 0.4 mg/ml and stored at – 80°C after reconstitution.

### ELISA

The binding IgG antibodies in immune sera were measured by ELISA. In brief, 96-well plates were coated with recombinant S or RBD proteins from Wuhan, Beta, Delta, or Omicron BA.1 strains (Sino Biological Inc., China) at 0.05 μg/well in 1 × phosphate-buffered saline (PBS) overnight at 4 °C, washed, and blocked with 1 × PBS supplemented with 0.05% Tween-20 (PBST) and 2% bovine serum albumin (BSA) for 2 h at room temperature. Serially diluted serum samples were added to each well and incubated for 2 h at 37°C. Subsequently, the wells were washed and added with horseradish peroxidase (HRP)-conjugated goat anti-monkey IgG antibodies (1:2000 in PBS containing 5% skim milk; Abcam). After incubation for 1 h at room temperature, the wells were washed and added with the 3,3’,5,5’-tetramethylbenzidine (TMB, ThermoFisher) substrate. Finally, the optical density was measured at 450 nm (OD_450_). The cutoff value was calculated as 2.1 times the OD_450_ values from the serum of non-vaccinated macaques at the same dilution. The endpoint titres were calculated as the reciprocal of the highest dilution at which the OD_450_ values were equal to or greater than the cutoff values.

### Neutralization assay for SARS-CoV-2

The NAb titres were quantified by neutralization assays based on S-pseudotyped vesicular stomatitis virus (VSV) according to a previously described method [22]. In brief, 293 T cells were transfected with plasmids expressing the S protein of SARS-CoV-2 Wuhan, Beta, Delta, or Omicron BA.1 strain. Meanwhile, the cells were infected with VSV of which the G gene was replaced with the firefly luciferase (Fluc) reporter gene. Thus, the S protein was incorporated onto the surface of the defective VSV. The pseudotyped reporter viruses could mimic the attachment and entry of authentic SARS-CoV-2. Serum samples were serially diluted 3-fold from 1:30–1:7290 with Dulbecco’s modified eagle’s medium (DMEM) supplemented with 1% penicillin–streptomycin (PS), 25 mM HEPES, and 10% fetal bovine serum (FBS). The diluted serum samples were added with pseudotyped viruses at 650 TCID50 per well and incubated at 37 °C for 1 h. The mixtures were then added into Huh-7 cells cultured in 96-well plates at 2 × 10^4^ cells per well, followed by incubation in 5% CO_2_ at 37˚C for 24 h. Finally, the luminescence was measured by Bright-GloTM luciferase detection reagent (Promega). The infection inhibition rates at each dilution were calculated according to the relative light unit (RLU) values as follows: inhibition rate = [1 – (average RLU of sample – average RLU of virus control) / (average RLU of virus control – average RLU of cell control)] × 100%. Based on the inhibition rate, the half maximal effective concentration (EC50) was calculated by Reed-Muench method.

### IFN-γ ELISpot

CMI responses against SARS-CoV-2 S protein or SIV antigens were measured by IFN-γ ELISpot assays according to a previously described method [23]. In brief, sterile 96-well microtiter plates (Merck Millipore) were coated with monkey IFN-γ coating antibody (U-CyTech) at 4 °C overnight. The plates were then blocked with R10 medium (RPMI 1640 supplemented with 10% FBS, 1% PS, 0.05 mM 2-mercaptoethanol, 1 mM sodium pyruvate, 2 mM L-glutamine, and 10 mM HEPES) at 37 °C for 2 h. PBMCs were isolated using a density gradient medium (LymphoprepTM, Canada) according to the manufacturer’s protocol, seeded in the plates at 3 × 10^5^ cells per well, and stimulated with each peptide pool at 2 μg/ml per peptide. DMSO and concanavalin A (ConA, 10μg/ml; Sigma) were also added as negative and positive controls, respectively. Subsequently, the plates were incubated at 37 °C in 5% CO_2_ for 24 h, washed with PBST, and then added with biotinylated detection antibodies (U-CyTech). After incubation at 4 °C overnight, the plates were washed with PBST, added with streptomycin-conjugated alkaline phosphatase (BD Pharmingen), and incubated at 37 °C for 2 h. Finally, the plates were washed and developed with pre-warmed BCIP/NBT substrate solution (Pierce). Spot counts and pictures were collected by an ELISpot imager (Bioreader 6000, BIOSYS, Germany). S-specific SFCs were calculated as the sum of those specific for S1 and S2.

### ASC ELISpot

Antigen-specific ASC ELISpot assays were performed according to a previously described method [24]. In brief, sterile 96-well plates (Merck Millipore) were coated with recombinant S or RBD proteins of SARS-CoV-2 Wuhan strain at 10 μg/ml overnight at 4°C. Subsequently, the plates were washed with PBS and blocked with R10 medium for 2 h at 37 °C. PBMCs in the R10 medium were seeded in the plates at 2 × 10^5^ cells per well and incubated overnight in a 5% CO_2_ incubator at 37 °C. The plates were then washed with PBST and added with horseradish peroxidase (HRP)-coupled rabbit anti-monkey IgG (H&L) antibodies (Immunoway). Finally, the plates were developed with an AEC reagent (BD Pharmigen). An ELISpot imager counted spots of ASCs. The frequencies of antigen-specific ASCs are reported as the number of spots per million cells.

### Memory B-cell ELISpot

Memory B-cell ELISpot assays were performed according to a previously described method [25]. In brief, PBMCs were seeded in 6-well plates at 3 × 10^6^ cells per well in an R10 medium supplemented with recombinant human IL-2 (100 U/ml, PeproTech) and R848 (1 μg/ml, InvivoGen), and cultured for 5 days in a 5% CO_2_ incubator at 37 °C. The stimulated PBMCs were then harvested, washed extensively with 1 × PBS, and subjected to ASC ELISpot assay as described above.

### Flow cytometry analysis

The numbers of circulating CD4^+^ T and CD8^+^ T lymphocytes were determined using BD Trucount absolute count tubes according to the manufacturer’s instructions (BD Biosciences). For detection of central memory CD4^+^ T (CD3 ^+ ^CD4 ^+ ^CD28 ^+ ^CD95 ^+ ^cells), effector memory CD4^+^ T (CD3 ^+ ^CD4 ^+ ^CD28^-^CD95^+^ cells) and TFH cell (CD3 ^+ ^CD4 ^+ ^CXCR5 ^+ ^PD-1^+^) cells, PBMCs were stained with the following fluorescein-labeled antibodies: anti-CD3 (BD Bioscience), anti-CD4 (BD Bioscience), anti-CD8 (BD Bioscience), anti-CD28 (BD Bioscience), anti-CD95 (BD Bioscience), anti-CXCR5 (eBioscience), and anti-PD-1 (eBioscience) for 30 min and detected with a BD FACS LSR Fortessa flow cytometer (BD Biosciences, USA). Data were analyzed using FlowJo software (Tree Star, USA).

### qRT-PCR

Plasma SIV viral loads were measured by one-step qRT-PCR. In brief, total RNA was extracted from the plasma samples using QIAamp Viral RNA Mini kit (Qiagen). The SIV genomic RNA was amplified and quantified using the QuantiTect SYBR Green RT–PCR Kit (Qiagen). The primer set includes the forward primer (5’ AATACTGTCTGCGTCATCTGG 3’), and the reverse primer (5’ ATGGTGCTGTTGGTCTACTTG 3’). The amplification procedures were set up as the following: 50 °C for 30 min, 95 °C for 15 min followed by 45 cycles consisting of 94 °C for 15 s, 60 °C for 30 s, and 72 °C for 30 s, and a default melting curve step in a Bio-Rad CFX96 Touch machine. The standard curve was generated using serial dilutions of SIV gag RNA fragments produced by in vitro transcription. The detection limit was determined by the standard curve and the dilution and was 100 copies per 1 ml plasma. The viral loads were calculated as the genome copies of SIV in one ml plasma.

### Complete blood count and blood biochemical test

In brief, the whole blood count was measured using XN-1000 V (Sysmex, Denmark), and the biochemical test was performed using 3100 (Hitachi, Japan), all procedures followed manual instructions and were operated by professional personnel.

### Neutralization assay for Ad5

NAbs against Ad5 vector were measured according to a previously described method [26]. In brief, human embryonic kidney 293 cells were seeded into 96-well plates and cultured for 24 h. Serum samples collected at week 0 were serially diluted after inactivation at 56 °C for 1.5 h and incubated with Ad5-SEAP which expresses secreted-alkaline-phosphatase reporter gene at 4 × 10^6^ vp per well at 37 °C for 1 h. The mixtures were then added to the 96-well plates and incubated in 5% CO_2_ at 37 °C for 24 h. Finally, the culture supernatants were harvested, and the SEAP activity was detected using a Phospha-Light System (Thermo Fisher). The NAb titres were calculated as dilutions that inhibited 50% RLU values.

### Statistics

Analysis of virological and immunological data was performed using GraphPad Prism version 7.0 (GraphPad Software). Comparisons between groups were conducted using an unpaired Students’ t-test (one-tailed). Comparisons between different time points in the same group were conducted using paired Students’ t-test (one-tailed). Differences were considered statistically significant when the *p* values were less than 0.05. Data graphs were constructed using GraphPad Prism version 7. Figures and illustrations were created using Photoshop version CS5 (Adobe Systems Inc.) and Powerpoint version 2010 (Microsoft).

## Results

### The magnitude and persistence of vaccine-induced antibody responses are impaired in macaques with severe SIV infection

**Figure 1.**
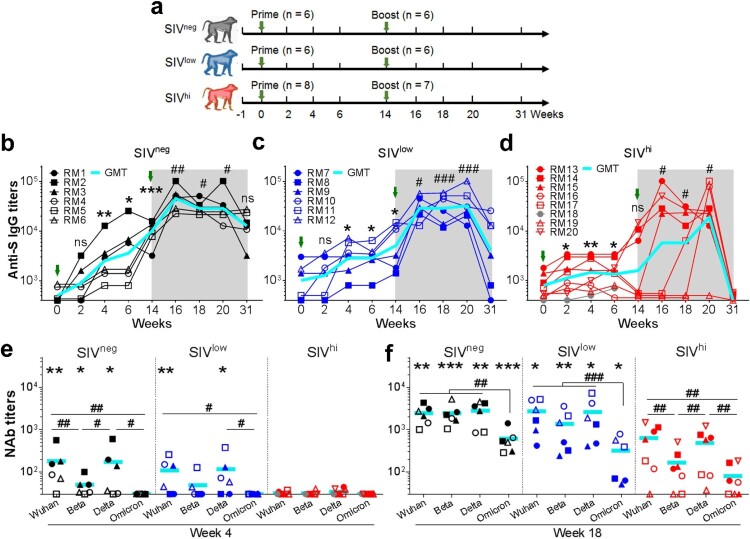
Anti-S_Wuhan_ IgG and NAb responses in macaques with mild or severe SIV infection. a. Schematic diagram of the timeline for vaccination and detection. Fourteen SIV-infected rhesus macaques not on ART were divided into SIV^low^ (n = 6; mean viral load: 3.4 log RNA copies per ml plasma; range: 2.0-4.7) and SIV^hi^ (n = 8; mean: 6.2; range: 5.0-7.7) groups. Six uninfected macaques were used as healthy controls (SIV^neg^). All the macaques received two intramuscular injections with 1 × 10^11^ vp of Ad5-S at weeks 0 and 14. At weeks 0, 2, 4, 6, 14, 16, 18, 20, and 31, serum samples and PBMCs were collected and subjected to virological and immunological analysis. Green arrows indicate the time points of vaccination. Major health indicators were monitored over the course. b-d. Kinetics of anti-S_Wuhan_ IgG antibody response in SIV^neg^ (b), SIV^low^ (c), and SIV^hi^ (d) macaques. The endpoint IgG titres against the S protein of the SARS-CoV-2 Wuhan strain are shown. The limit of detection is 1:400. Overlapped data points represent the same values. Comparisons were conducted between week 2, 4, 6, 14 and week 0 (*, **, ***) or between week 16, 18, 20, 31 and week 14 (#, ##, ###) by paired, one-tailed Student’s t-test. Green arrows indicate the time points of vaccination. The shaded grey area indicates the period after the booster vaccination. Macaque RM18 died at week 8 due to severe SIV infection and was marked as gray dots. e, f. NAb responses against Wuhan strain and VOCs in each group at week 4 (e) and week 18 (f). S-pseudotyped vesicular stomatitis viruses assessed serum samples. The limit of detection is 1:30. Comparisons of the NAb titres against each strain in the same group were conducted by paired, one-tailed Student’s t-test (#, ##, ###). Comparisons of the NAb titres against the same strain in SIV^neg^ or SIV^low^ group versus SIV^hi^ group were conducted by unpaired, one-tailed Student’s t-test (*, **, ***). All the data points represent the mean values of two technical replicates. * or #, *p* < 0.05; ** or ##, *p* < 0.01; *** or ###, *p* < 0.001; ns, no significance.

A total of 20 macaques were included, among whom 14 macaques had chronic SIVmac239 infection for more than 2 years (Table S1). Plasma viral loads in SIV-infected macaques reached a stable setpoint 5 months after discontinuation of ART, which resembles PLWH without ART or with ART failure. High viral load is defined as 5 log RNA copies per ml plasma based on literature review [27,28]. The macaques with high setpoint SIV viral loads (SIV^hi^; n = 8; mean: 6.2 log RNA copies per ml plasma; range: 5.0-7.7) had a greater loss of CD4^+^ T cells (mean: 776 per μl blood; range: 236-1360) and TFH cells and inverted CD4/CD8 ratios, showing severe immunodeficiency or “severe SIV infection”, as compared to those with low setpoint viral loads (SIV^low^; n = 6; mean: 3.4; range: 2.0-4.7) and higher CD4^+^ T cells (mean: 1316; range: 954-2214) (Figure S1), or “mild SIV infection”. Macaques without SIV infection (SIV^neg^; n = 6) were used as healthy controls with mean CD4^+^ T cell counts at 1761 (range: 1155-3711). Each animal received two intramuscular vaccinations with Ad5-S at 1 × 10^11^ viral particles (vp) at a 14-week interval (Figure 1a). One SIV^hi^ macaque (RM18) died at week 8 due to SIV-associated anemia and myocardial injury, of whom the data after week 8 were unavailable.

**Figure 2.**
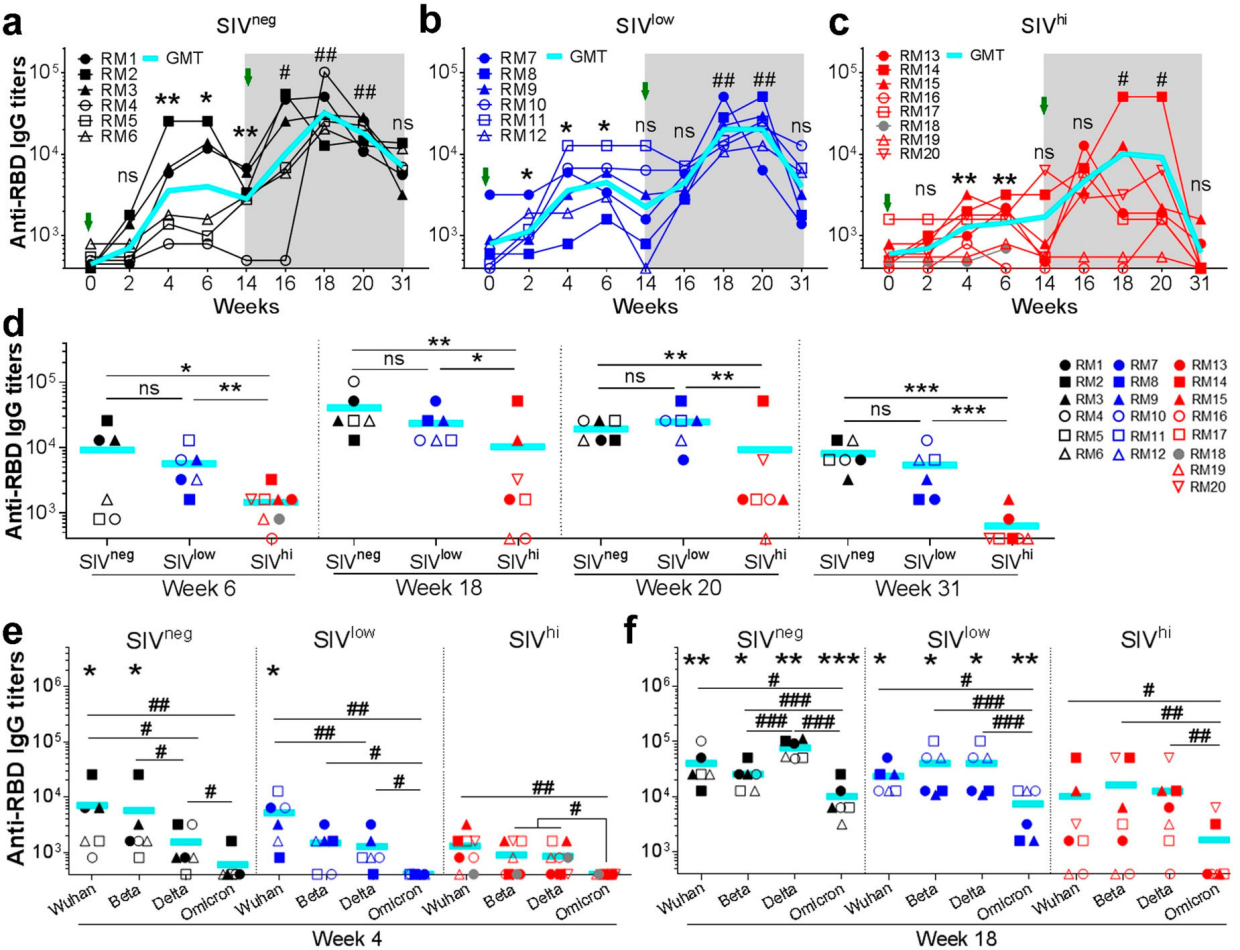
Anti-RBD_Wuhan_ IgG responses in macaques with mild or severe SIV infection. a-c. kinetics of anti-RBD_Wuhan_ IgG titres in SIV^neg^ (a), SIV^low^ (b), and SIV^hi^ (c) macaques. The endpoint IgG titres against SARS-CoV-2 Wuhan strain are shown. The limit of detection is 1:400. Overlapped data points represent the same values. Comparisons were conducted between weeks 2, 4, 6, 14, and week 0 (*, **) or between weeks 16, 18, 20, 31, and week 14 (#, ##) by paired, one-tailed Student’s t-test. Green arrows indicate the time points of vaccination. The shaded grey area indicates the period after the booster vaccination. d. Comparisons of the anti-RBD_Wuhan_ IgG titres between groups at weeks 6, 18, 20, and 31. Comparisons were performed by unpaired, one-tailed Student’s t-test. e, f. Anti-RBD IgG responses against Wuhan strain and VOCs in each group at week 4 (e) and week 18 (f). Comparisons of the anti-RBD IgG titres against each strain in the same group were conducted by paired, one-tailed Student’s t-test (#, ##, ###). Comparisons of the anti-RBD IgG titres against the same strain in SIV^neg^ or SIV^low^ group versus SIV^hi^ group were conducted by unpaired, one-tailed Student’s t-test (*, **, ***). All the data points represent the mean values of two technical replicates. * or #, *p* < 0.05; ** or ##, *p* < 0.01; *** or ###, *p* < 0.001; ns, no significance.

A single vaccination with Ad5-S elicited anti-S_Wuhan_ IgG antibody response in all SIV^neg^ and 4 out of 6 SIV^low^ macaques at week 4. The geometric mean titre (GMT) in these two groups continuously increased until week 14. A booster vaccination further increased the response in SIV^neg^ (4.5 folds) and SIV^low^ (5.7 folds) groups, which persisted for at least 17 weeks in all SIV^neg^ and 5 out of 6 SIV^low^ animals (Figure 1b and c). In contrast, a single vaccination elicited only a moderate response in 5 out of 8 SIV^hi^ animals at week 4. The titres rapidly declined to be undetectable in 4 out of 7 SIV^hi^ animals by week 14 (Figure 1d). A booster vaccination enhanced anti-S_Wuhan_ IgG titres in 6 out of 7 SIV^hi^ animals, albeit delayed in macaques RM16 and RM17 and without response in macaque RM19. The titres rapidly declined to be undetectable in SIV^hi^ animals by week 31 (Figure 1d). The anti-S_Wuhan_ IgG titres in SIV^neg^ and SIV^low^ groups were comparable, and both were higher than those in SIV^hi^ group (Figure S2). Thus, severe SIV infection impacts the magnitude and persistence of the anti-S_Wuhan_ IgG antibody response elicited by Ad5-S. A booster vaccination is beneficial for SIV^hi^ macaques to mount this response. Anti-RBD_Wuhan_ IgG antibody response was detectable in all SIV^neg^ and SIV^low^ macaques after one vaccination. A booster vaccination greatly enhanced it by 11.3 and 9.0 folds, respectively (Figure 2a and b). Two SIV^hi^ macaques (RM16 and RM19) showed no response after the first vaccination, a booster vaccination moderately elevated the anti-RBD_Wuhan_ IgG antibody response in 5 out of 7 SIV^hi^ macaques (Figure 2c). The anti-RBD_Wuhan_ IgG titres in SIV^hi^ group declined to nearly undetectable levels, whereas they persisted in SIV^neg^ and SIV^low^ groups for at least 17 weeks after the booster vaccination (Figure 2d).

We also assessed anti-RBD IgG antibody responses against VOCs Beta, Delta, and Omicron (Figure 2e). After a single vaccination, anti-RBD_Beta_ IgG and anti-RBD_Delta_ IgG but not anti-RBD_Omicron_ IgG were detectable in most SIV^neg^ and SIV^low^ macaques (Figure 2e). A booster vaccination significantly improved the anti-RBD IgG titres against Beta, Delta, and Omicron in all SIV^neg^ and SIV^low^ macaques. In SIV^hi^ group, 5 out of 7, 5 out of 7, 6 out of 7, and 2 out of 7 macaques mounted anti-RBD_Wuhan_, anti-RBD_Beta_, anti-RBD_Delta_, and anti-RBD_Omicron_ IgG, respectively (Figure 2f). Hence, although severe SIV infection impairs the magnitude and breadth of anti-RBD IgG response elicited by Ad5-S, a booster vaccination is beneficial for SIV-infected macaques to mount cross-reactive antibody responses against VOCs.

We next tested the NAb responses via a neutralization assay based on S-pseudotyped vesicular stomatitis viruses. A single vaccination elicited NAbs against Wuhan strain in 5 out of 6 SIV^neg^ macaques, among whom 3 animals also had NAbs against Beta and Delta (Figure 1e) but not Omicron. A single vaccination elicited NAbs against Wuhan and Delta in 4 out of 6 SIV^low^ macaques, and Beta in 2 out of 6 SIV^low^ macaques. By contrast, a single vaccination elicited no NAbs in any SIV^hi^ macaques (Figure 1e). Intriguingly, a booster vaccination significantly improved the NAb titres in all SIV^neg^, SIV^low^, and SIV^hi^ macaques against Wuhan (18.5, 24.9, and 11.0 folds, respectively), Beta (49.5, 21.4, and 3.4 folds, respectively), and Delta (25.2, 20.8, and 7.8 folds, respectively). NAb titres against Omicron were elevated in all SIV^neg^ (17.6 folds) and SIV^low^ (6.1 folds) macaques and in 4 out of 7 SIV^hi^ macaques (Figure 1f). However, the average NAb titres against Wuhan, Beta, Delta, and Omicron in SIV^hi^ macaques were 6.4, 20.2, 8.6, and 8.8 folds lower than those in SIV^neg^ macaques, respectively (Figure 1f). Thus, severe SIV infection impairs the magnitude and breadth of NAb responses elicited by Ad5-S. A booster vaccination can significantly enhance this response in SIV^low^ macaques but less in SIV^hi^ macaques.

### Vaccine-induced antibody responses are associated with baseline SIV viral loads and the TFH and Tcm subsets of CD4^+^ T cells

**Figure 3.**
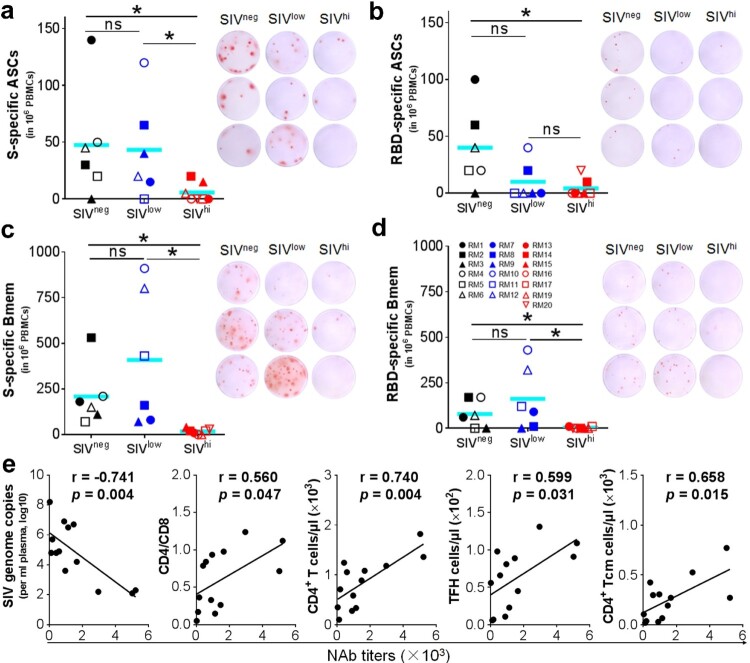
SARS-CoV-2 specific B cell responses, and correlation analysis of NAb titres versus SIV viral loads and CD4^+^ T cell populations. a, b. Antibody-secreting B cells (ASCs) specific for S_Wuhan_ (a) and RBD_Wuhan_ (b) in PBMCs collected at 4 days after the booster vaccination. PBMCs were stimulated with recombinant S or RBD proteins of SARS-CoV-2 Wuhan strain. IgG-secreting B cells were measured by ELISpot. c, d. Memory B cells (Bmem) specific for S_Wuhan_ (c) and RBD_Wuhan_ (d) in PBMCs were collected at 6 weeks after the booster vaccination (week 20). PBMCs were incubated with recombinant human IL-2 and R848 for 5 days followed by stimulation with recombinant S or RBD proteins of SARS-CoV-2 Wuhan strain. The IgG-secreting memory B cells were measured by ELISpot. The frequencies (left) and representative wells (right) are shown. Comparisons between groups were performed by unpaired, one-tailed Student’s t-test. *, *p* < 0.05; ns, no significance. e. Correlations between the NAb titres at week 18 versus the virological and immunological parameters (left to right: SIV viral loads, CD4/CD8 ratios, the counts of total CD4^+^ T cells, TFH cells, and CD4^+^ Tcm cells) at week 14. The best fit lines, r values, and *p* values are shown.

We analyzed the factors that may influence the ability of Ad5-S to induce antibody responses. Anti-RBD IgG titres at week 6 negatively correlated to baseline SIV viral loads (r = −0.740, *p *= 0.003) and positively to the counts of TFH cells at week 0 (r = 0.540, *p *= 0.046) (Figure S3), suggesting that CD4^+^ T cells, in particular the TFH subset, may be important for the generation of anti-RBD IgG antibodies. Because the magnitude of antibody responses is associated with the frequency of antibody-secreting B cells (ASCs) [29], we tested the ASCs specific for S_Wuhan_ in the peripheral blood mononuclear cells (PBMCs) collected at 4 days after the booster vaccination by enzyme-linked spot-forming test (ELISpot). S-specific ASCs were detected in 5 out of 6 SIV^neg^ and SIV^low^ but in only 2 out of 7 SIV^hi^ macaques (Figure 3a). RBD-specific ASCs were detected in 5 out of 6 SIV^neg^ but only in 2 out of 6 SIV^low^ and in 1 out of 7 SIV^hi^ macaques (Figure 3b). To test the memory B (Bmem) cells elicited by vaccination, we performed memory B-cell ELISpot using the PBMCs collected at week 20. S-specific Bmem cells were detected in all SIV^neg^ and SIV^low^ but not in SIV^hi^ macaques (Figure 3c). RBD-specific Bmem cells were detected in 4 out of 6 SIV^neg^ and SIV^low^ but not in SIV^hi^ macaques (Figure 3d). Notably, S-specific Bmem cells negatively correlated to the SIV viral loads (r = −0.785, *p *= 0.002) at booster vaccination (week 14), but positively correlated to the counts of CD4^+^ T (r = 0.746, *p *= 0.003), TFH (r = 0.640, *p *= 0.018) and CD4^+^ Tcm cells (r = 0.840, *p *< 0.001), and the CD4/CD8 ratios (r = 0.571, *p *= 0.041) at week 14. RBD-specific Bmem cells also negatively correlated to the viral loads (r = −0.707, *p *= 0.007) at week 14. but positively to the counts of CD4^+^ T (r = 0.732, *p *= 0.005), TFH (r = 0.596, *p *= 0.032) and CD4^+^ Tcm cells (r = 0.864, *p *< 0.001) (Figure S4). Interestingly, the frequencies of S – and RBD-specific Bmem cells positively correlated to the anti-S and anti-RBD IgG titres at week 31, respectively (Figure S5), implying that the durability of antibody responses may be associated with the memory B cell responses. We also analyzed SARS-CoV-2 NAb titres (Wuhan) 4 weeks after the booster vaccination. The NAb titres negatively correlated to the viral loads (r = −0.741, *p *= 0.004) at week 14, but positively correlated to the counts of CD4^+^ T (r = 0.740, *p *= 0.004), TFH (r = 0.599, *p *= 0.031) and CD4^+^ Tcm (r = 0.658, *p *= 0.015) cells, and the CD4/CD8 ratios (r = 0.560, *p *= 0.047) (Figure 3e, Figure S6), implying that CD4^+^ T cells, in particular the TFH and Tcm subsets, are critical for the generation of NAbs. Collectively, severe SIV infection significantly destroys CD4^+^ T cell populations, especially the TFH and Tcm subsets, which may disable the effector and memory B cell responses, leading to the impairment of the antibody responses elicited by a COVID-19 vaccine.

### Vaccine-induced CMI response is impaired in macaques with severe SIV infection and is associated with baseline SIV viral loads and CD4/CD8 ratios

**Figure 4.**
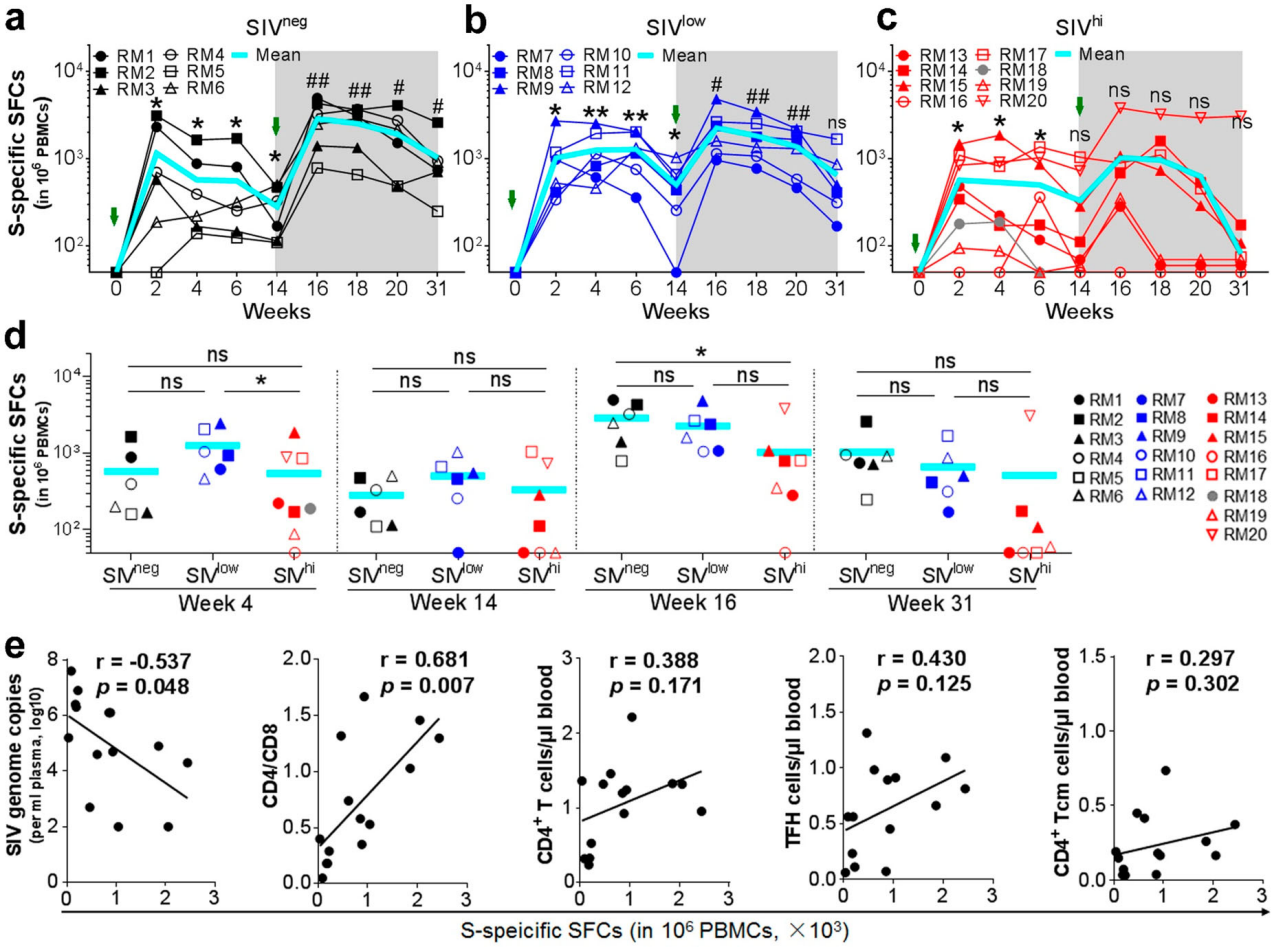
S_Wuhan_-specific CMI responses in macaques with mild or severe SIV infection. a-c. Kinetics of S_Wuhan_-specific IFN-γ-secreting cells in SIV^neg^ (a), SIV^low^ (b), and SIV^hi^ (c) macaques. PBMCs were collected at the indicated time points and stimulated with overlapped peptide pools corresponding to the S1 and S2 subunits of the S protein of SARS-CoV-2 Wuhan strain. IFN-γ-secreting spot-forming cells (SFCs) were examined by ELISpot. S-specific SFCs were calculated as the sum of those specific for S1 and S2. Overlapped data points represent the same values. A Shaded grey area indicates the period after the booster vaccination. Comparisons were conducted between weeks 2, 4, 6, 14, and week 0 (*, **) or between weeks 16, 18, 20, 31, and week 14 (#, ##) by paired, one-tailed Student’s t-test. The mean values and statistics in (c) were calculated without the data from the outlier macaque RM20. d. Frequencies of IFN-γ-secreting SFCs in each group at weeks 4, 14, 16, and 31. Comparisons between groups at each time point were performed by unpaired, one-tailed Student’s t-test. Mean values and statistics at weeks 16 and 31 were calculated without the data from the outlier macaque RM20. **e.** Correlations between the frequencies of S_Wuhan_-specific SFCs at week 4 versus the virological and immunological parameters (left to right: SIV viral loads, CD4/CD8 ratios, the counts of total CD4^+^ T cells, TFH cells, and CD4^+^ Tcm cells) at week 0. The best fit lines, r values, and *p* values are shown. * or #, *p* < 0.05; ** or ##, *p* < 0.01; ns, no significance.

Besides antibody response, CMI response also contributes to protection against SARS-CoV-2 infection [30]. We tested the IFN-γ-secreting cells in the PBMCs by ELISpot using overlapping peptide pools covering the S protein of Wuhan strain. A single vaccination elicited S-specific IFN-γ^+^ spot-forming cells (SFCs) in all SIV^neg^ and SIV^low^ macaques (Figure 3a and b). The frequencies of IFN-γ^+^ SFCs peaked at week 2 (mean SFC: 1154 and 1027), elevated again by a booster vaccination (mean SFC: 2865 and 2264), and remained at a relatively high level by week 31 (mean SFC: 905 and 648) (Figure 4a, b, d). In contrast, the CMI response rapidly declined to undetectable in 3 out of 7 SIV^hi^ animals at week 14 after one vaccination. Although the CMI response can be enhanced at 2 weeks after the booster vaccination, again, it rapidly declined to be nearly undetectable in 5 out of 7 SIV^hi^ animals at week 31 (Figure 4c), except for one SIV^hi^ macaque (RM20) (Figure 4d). Excluding this outlier, the CMI response in SIV^hi^ group was significantly lower (mean SFC: 558) than that in both SIV^low^ and SIV^neg^ groups at week 16 (*p* = 0.009 and 0.003, respectively) and week 31 (*p* = 0.015 and 0.009, respectively). We also used intracellular cytokine staining assay (ICS) to quantify cytokine-producing antigen-specific T cells at week 4. We found that IFN-γ-producing CD4^+^ T cells and ELISpot (IFN-γ-producing PBMCs) have similar expression patterns at week 4 (Figure S7). The CMI response at week 4 negatively correlated to baseline SIV viral loads (r = −0.537, *p *= 0.048) but positively to the CD4/CD8 ratio (r = 0.681, *p *= 0.007) (Figure 4e, Figure S8). Thus, a booster vaccination can enhance CMI response in both SIV^low^ and SIV^high^ macaques, but severe SIV infection impairs the magnitude and persistence of S-specific CMI response.

### Pre-existing anti-Ad5 NAbs have no significant impact on the immunogenicity of Ad5-S in SIV-infected macaques

Pre-existing anti-Ad5 NAbs usually have high seroprevalence in populations and may dampen the immunogenicity of Ad5-vectored vaccines [31]. We analyzed the correlation between antibody titres or CMI responses and pre-existing anti-Ad5 NAbs in SIV^neg^ and SIV^low^ macaques. Anti-S IgG titres, NAb titres, and CMI response after a single vaccination showed no significant correlation to the pre-existing anti-Ad5 NAbs at week 0. After a booster vaccination, anti-S IgG titres, NAb titres, and CMI response showed no significant correlation to the increased anti-Ad5 NAbs at week 14 (Figure S9). Thus, at the tested vaccination dosage, a high level of pre-existing anti-Ad5 NAbs showed a trend but no significant attenuation to the immunogenicity of Ad5-S.

### Ad5-vectored COVID-19 vaccination does not exacerbate the existing SIV infection or the associated diseases

The vaccination itself can activate various immune cells, raising the concern that administration of Ad5-S may affect SIV replication [4]. We found that the SIV viral loads remained relatively stable after either one or two vaccinations (Figure S10a). Thus, vaccination using the Ad5-vectored COVID-19 vaccine is unlikely to promote SIV replication. There were no signs of adverse effects after either one or two vaccinations. Blood count and blood biochemistry showed no abnormality in vaccinated macaques (Table S2). The counts of peripheral CD4^+^ or CD8^+^ T cells or the CD4/CD8 ratios remained relatively stable, except for a few transient fluctuations at several time points (Figure S10b-d). Hence, Ad5-S vaccination is unlikely to exacerbate the SIV-caused immunodeficiency. We also monitored CMI response against SIV antigens. The frequencies of IFN-γ^+^ SFCs specific for SIV Gag and Pol maintained similar levels or had slight fluctuations before and after vaccination (Figure S10e and f).

## Discussion

PLWH with immunosuppression may be a source of SARS-CoV-2 transmission and the emergence of variants [11]. PLWH should be prioritized for receiving COVID-19 vaccination for better control of SARS-CoV-2 spread and the occurrence of VOCs. Our study evaluated if an Ad5-vectored COVID-19 vaccine is immunogenic in severe or mild immunosuppression, in the absence of ART. This may have significance for the application of COVID-19 vaccine in PLWH not receiving ART or experiencing ART failure. Like ChAdOx1 nCoV-19 and BNT162b2 vaccines that effectively elicit antibody and CMI responses in PLWH on ART and with CD4^+^ T-cell counts greater than 350 [4,5], Ad5-S elicits such responses in SIV^low^ macaques with relatively high CD4^+^ T-cell counts. One dose of BNT162b2 mRNA vaccine elicits a low anti-RBD IgG response in PLWH with CD4^+^ T-cell counts less than 200 [32], also similar to our observation that one dose of Ad5-S elicits a lower antibody and CMI responses in SIV^hi^ macaques that have lower CD4^+^ T-cell counts. Decreased immunogenicity in patients with low CD4^+^ T-cell counts or uncontrolled viremia has also been observed for vaccines against other pathogens such as influenza virus, Hepatitis A or B virus, as well as pneumococcus [33]. Nevertheless, it is still possible to provoke effective immunity against SARS-CoV-2 by a booster vaccination in all SIV^low^ animals and most SIV^hi^ animals. Notably, a booster vaccination facilitates the generation of cross-reactive NAbs against VOCs, including the highly contagious Delta and Omicron, which has also been observed in macaques received three doses of mRNA-based vaccine [34]. The immunogenicity of an Ad5-vectored COVID-19 vaccine is impaired by severe SIV infection. It can be partially improved by a booster vaccination, revealing the feasibility of building immune barriers in PLWH, even in the absence of ART.

Besides CD4^+^ T-cell depletion, HIV-1 infection also causes defective B-cell responses due to the loss of proper CD4^+^ T-cell responses [35,36]. The positive correlations between IgG and NAb titres versus the total CD4^+^ T cells or the TFH or Tcm subsets reveal that SIV infection perturbs these cells and impacts vaccination-elicited antibody responses. Diminished S – and RBD-specific ASCs in SIV^hi^ macaques imply that severe SIV infection seriously damages CD4^+^ T-cell populations, which weakens B cells’ ability to respond to antigen stimuli [35]. In contrast, SIV^low^ macaques have nearly normal ASCs, implying that mild SIV infection, even in the absence of ART, does not severely disturb CD4^+^ T-cell function. The B cells respond well to vaccination or natural infection. Indeed, patients with HIV-1 viral loads well-controlled under ART have fair responses against COVID-19 vaccination or SARS-CoV-2 infection [4,5]. Moreover, severe SIV infection causes defects in memory B cells [35,37]. Since memory B cells can differentiate into ASCs in a T help-dependent manner when stimulated by antigens, they are crucial for the rapidity and durability of antibody responses [38,39]. The defects of these cells predict limited long-term responses, as evidenced by the poor persistence of antibody response in SIV^hi^ macaques. In PLWH whose CD4^+^ T cells have been severely destroyed, the antibody responses against SARS-CoV-2 should be cautiously monitored. Booster vaccinations may be frequently required to maintain effective immunity.

In the SIV^low^ group, the antibody level of 3 macaques (RM7, 8, 9) showed a late decline, while the other 3 macaques (RM10, 11, 12) showed no significant decline at booster vaccination. We observed that 3 macaques (RM7, 8, 9) had significantly higher SIV loads, and lower average CD4^+^ T and circulation TFH cell counts than the other 3 macaques (RM10, 11, 12). It appears that SIV infection causes damage to CD4^+^ T cell population, which in turn impacts the generation of memory B cells and long-term antibody responses.

In this study, we observed that the frequencies of S-specific and RBD-specific Bmem cells in a proportion of SIV^low^ macaques (RM10, 11, 12) were slightly higher compared to SIV^neg^ macaques. However, there is no significant difference between SIV^low^ and SIV^neg^ macaques in the overall level of Bmem cell response. This seems to be consistent with the comparable antibody responses between SIV^low^ and SIV^neg^ macaques. This may be associated with the relatively high frequencies of circulating TFH cells, which are critical for the generation of antibody and Bmem responses [14,40]. In addition, the SIV^low^ macaques are usually experiencing a continuous immune activation status, and thus prone to generate memory B cells and transient ASC upon antigen stimuli [35,41].

Our study also added to the knowledge about the CMI responses elicited by an Ad-vectored COVID-19 vaccine, which remains obscure in untreated PLWH. Similar to ChAdOx1 nCoV-19 which effectively elicits S-specific CMI responses in well-managed PLWH [4], Ad5-S elicits strong S-specific CMI responses in SIV^low^ macaques, revealing the possibility that Ad-vectored COVID-19 vaccines can provoke CMI responses in patients with mild HIV-1 infection, even in the absence of ART. This is of great significance because CMI response is mostly cross-reactive and long-lasting and thus contributes to protection against VOCs. In contrast, the protective efficacy of antibody responses can be eliminated along with the mutations in S protein, as observed for Omicron [42]. However, severe SIV infection impairs the magnitude and durability of CMI responses elicited by Ad5-S, raising the concern that CMI response cannot be effectively provoked in some patients with advanced HIV-1 infection. Poor CMI response correlates to high SIV viral loads and inverted CD4/CD8 ratios, implying that severe SIV infection causes immunosenescence that disturbs the generation of CMI responses [43–45]. Immune reconstitution via ART may be beneficial before administering an Ad-vectored COVID-19 vaccine in untreated PLWH.

The genetic background of macaques is very complex, so individual differences extensively exist for macaque study, which leads to the fluctuation of experimental data. However, this status can also better mimic the complex genetic diversity for human population. In this study, we observed that one SIV^hi^ macaque (RM20) has quite good and persistent T cell response. It is not yet possible to identify a plausible reason for the outlier (RM20). However, such outstanding responders have also been reported in other studies. An acute-phase study of four Mamu-B*08^+^ macaques found that three macaques controlled their viral set points of SIVmac239 replication below 2 × 10^4^ copies/ml, while one (r91003) had a viral load as high as 5 × 10^5^ copies/ml. However, macaque r91003 made a more robust immune response against the CD8^+^ T cell epitopes Gag_181-189_ and Tat_28-35_ [46].

Flow cytometry-based quantification and analysis is help to clarify the phenotypes of vaccination-induced T and B cells. In this study, we performed ELISpot assays but not flow cytometry analysis after week 4 because frequent blood sampling, especially in a large volume, might be harmful to the wellbeing of SIV-infected macaques. Moreover, an ELISpot assay may be better than flow cytometry analysis in that it not only measures the frequency of antigen-specific cells but also determines if they are functional. In addition, for measuring antigen-specific T or B cells, of which the frequencies are usually low, the ELISpot assay may be a better choice due to its lower detection limit [47]. One previous study showed that antigen-specific cells represent only 1-2% of circulating IgG^+^ memory B cells (0.015-0.03% of PBMC) after recent vaccination [48]. This frequency may be too low to be detectable for flow cytometry.

We noted that a challenge study would directly demonstrate the protective effects of COVID-19 vaccination in SIV-infected macaques. However, the challenge assay with live SARS-CoV-2 must be carried out in a qualified Animal Biosafety Level 3 (ABSL-3) lab, which is currently unavailable in our institute or other local institutes. It is also infeasible to transport the SIV-infected macaques to the ABSL-3 labs in other regions. Nevertheless, the neutralizing antibody responses and antigen-specific T cell responses may be used as surrogates for predicting protective efficacy. A variety of recent studies have shown that the neutralizing antibody titres measured by the S-pseudotyped VSV-based neutralization assay, which was also used in our study, are well correlated with the protective efficacy of COVID-19 vaccines in both animal models and humans [13,49,50]. Antigen-specific cell-mediated immune responses have also been shown to confer cross-reactive protection against SARS-CoV-2 in rhesus macaques [51]. Thus, a challenge study is the best for comprehensively assessing the protective efficacy but may be not essential for clarifying the immunogenicity of a COVID-19 vaccine in the context of SIV-caused immunodeficiency.

In this study, there are 1–2 more female macaques in SIV^neg^ group (3/6) than in SIV^low^ (1/6) and SIV^hi^ groups (2/8). However, we did not observe any significant difference between gender groups in the anti-S IgG titres, NAb titres, or CMI responses after either a single or the booster vaccination. We noted that slightly longer years of SIV infection in SIV^hi^ macaques (mean: 3.9 years, range: 2.5-12 years) compared to SIV^low^ macaques (mean: 2.8 years, range: 2.1-4.5 years). However, there is no significant difference between SIV^low^ and SIV^hi^ macaques in the years of SIV infection. We also did not observe the correlation between antibody titres or CMI responses in SIV-infected macaques and the duration of SIV infection.

We observed that 3 SIV^low^ macaques experienced a transient increase of SIV load after one vaccination. However, there is no significant change in the SIV loads after one vaccination compared with pre-vaccination. A similar phenomenon has been found in some PLWHs (3/10), with a transient increase in HIV plasma viral load following hepatitis B vaccination [52]. We speculated that the transient increase of SIV loads and decline thereafter in these 3 macaques reflected the immune activation caused by vaccination and the immune control of SIV replication. The absence of such transient increase of SIV loads after the second vaccination may also be attributed to the anti-SIV immune responses provoked by initial SIV replication and immune activation by vaccination.

In summary, we demonstrated that SIV-infected macaques could mount good antibody and CMI responses with two dosages of vaccination using an Ad5-vectored COVID-19 vaccine. However, the magnitude, breadth, and duration of antibody response were attenuated by severe SIV infection, which more frequent booster vaccinations may overcome. The findings that vaccination-elicited antibody and CMI responses correlate to baseline viral loads, TFH counts, and CD4/CD8 ratios have implications for predicting COVID-19 vaccine efficacy in PLWH.

## Supplementary Material

Supplemental Material

## Study approval

All animal experiments were approved by the Institutional Animal Care and Use Committee of Guangzhou Institutes of Biomedicine and Health (GIBH), Chinese Academy of Sciences (IACUC No. N2021101).
